# The Mammalian Class 3 PI3K (PIK3C3) Is Required for Early Embryogenesis and Cell Proliferation

**DOI:** 10.1371/journal.pone.0016358

**Published:** 2011-01-20

**Authors:** Xiang Zhou, Jun Takatoh, Fan Wang

**Affiliations:** 1 Department of Cell Biology, Duke University Medical Center, Durham, North Carolina, United States of America; 2 Department of Neurobiology, Duke University Medical Center, Durham, North Carolina, United States of America; University of Dayton, United States of America

## Abstract

The *Pik3c3* gene encodes an 887 amino acid lipid kinase, phosphoinositide-3-kinase class 3 (PIK3C3). PIK3C3 is known to regulate various intracellular membrane trafficking events. However, little is known about its functions during early embryogenesis in mammals. To investigate the function of PIK3C3 *in vivo*, we generated *Pik3c3* null mice. We show here that *Pik3c3* heterozygous are normal and fertile. In contrast, *Pik3c3* homozygous mutants are embryonic lethal and die between E7.5 and E8.5 of embryogenesis. Mutant embryos are poorly developed with no evidence of mesoderm formation, and suffer from severely reduced cell proliferations. Cell proliferation defect is also evident *in vitro*, where mutant blastocysts in culture fail to give rise to typical colonies formed by inner cell mass. Electron microscopic analysis revealed that epiblast cells in mutant embryos appear normal, whereas the visceral endoderm cells contain larger vesicles inside the lipid droplets. Finally, we provide evidence that mTOR signaling is drastically reduced in *Pik3c3* null embryos, which could be a major contributor to the observed proliferation and embryogenesis defects.

## Introduction

In mammals, there are three classes of phosphoinositide-3-kinases (PI3Ks). Most of the previous studies on the *in vivo* role of PI3Ks focused on class I PI3Ks, which were shown to be required for early embryonic development [1], [2], [3]. However, the role of the class III PI3K, PIK3C3 (also called Vps34), during mammalian development is not known. PIK3C3/Vps34 represents the most ancient form of PI3Ks and is the only PI3K in yeast [4]. Previous studies have shown that PIK3C3 regulates several membrane trafficking events including homotypical fusion between early endosomes [5], [6], [7], bi-directional transportation of early endosomes along the microtubules [8], [9], maturation of endosomes or phagosomes [10], [11], [12], [13], biogenesis of multivesicular bodies [14], [15] and retrograde transportation from endosomes to the Golgi apparatus [16], [17]. PIK3C3 is also critical for the formation of pre-autophagosomes which are important for degradation of large protein aggregates and organelles [18], [19], [20], [21], [22]. Finally, PIK3C3 is shown to be required for nutrient/amino acid mediated activation of mTOR signaling in cultured cells [23], [24], [25], [26].

Deletion phenotypes of *Pik3c3/vps34* have been analyzed in several yeast and invertebrate organisms. In *Saccharomyces cerevisiae*, mutation in the *Vps34* gene results in mis-sorting and secretion of Golgi-modified precursor forms of several vacuolar hydrolases [27]. It also results in temperature-sensitive growth defects, osmo-regulation defects, vacuole segregation defects during mitosis [28], as well as a disruption of macroautophagy [20]. In *Caenorhabditis elegans*, *Pik3c3* mutation arrests worm development at the L3-L4 molt [29]. In *Drosophila melanogaster*, deletion of *Pik3c3* gene causes hemizygous larval lethality, failure in autophagosome formation and defective endocytosis in fat body cells. However, TOR signaling is not affected in *Pik3c3* mutant flies [30]. In mice, conditional knockout of *Pik3c3* in postmitotic sensory neurons results in fast and differential neurodegeneration caused by a severe disruption of the endosomal pathway [31], [32].

Here, we generated a null allele of *Pik3c3* in mouse. We found that homozygous mutant embryos are poorly developed, die at the beginning of gastrulation, and show significantly reduced cell proliferation rate both *in vivo* and *in vitro*. We also examined the ultrastructure and mTOR signaling in *Pik3c3* mutant embryos. Our study provides new insights into the importance of PI3KC3 for normal cell proliferation and embryogenesis.

## Results

### Generation of *Pik3c3* knockout mice

**Figure 1:**
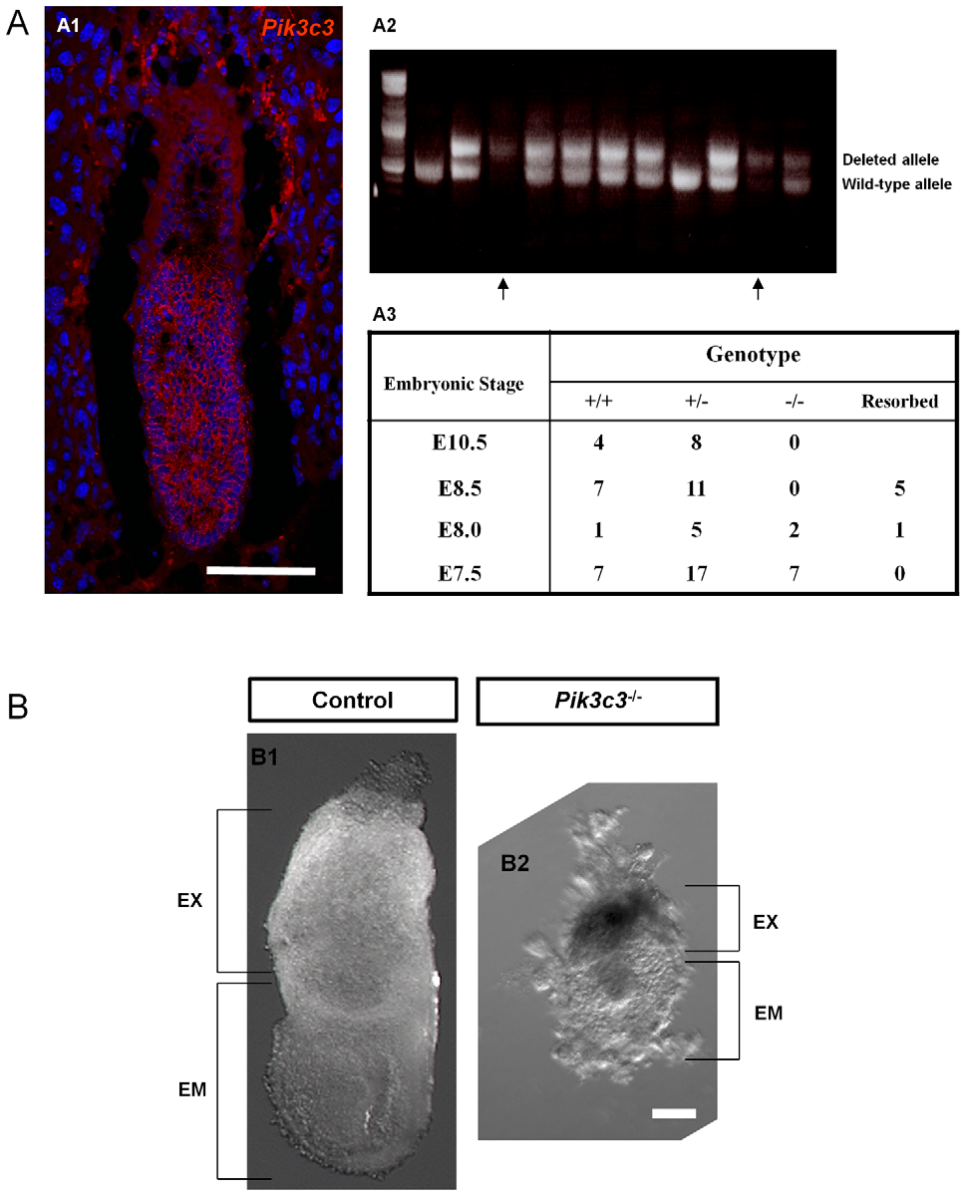
*Pik3c3* null mutant mice are embryonic lethal. **A.** A1: fluorescence in situ hybridization detected *Pik3c3* mRNA expression (red) in embryo at E6.5, counter stained with DAPI (blue). A2: representative PCR genotyping result of one litter at E7.5 from heterozygous intercrosses. The deleted allele produces a 670 bp fragment while the wild type allele produces a 450 bp fragment. A3: genotyping analysis of offspring from heterozygous intercrosses at different embryonic stages. Most mutant embryos are dying after around E7.5. **B.** Representative wholemount preparation of a heterozygous control (B1) and a homozygous mutant (B2) embryo at E7.5. Genotypes were obtained by PCR. Mutant embryos are smaller than controls. EX: extraembryonic region. EM: embryonic region. Scale bars: A, B: 100 µm.

We first examined the expression pattern of the *Pik3c3* gene in early embryos. Previous studies have shown that *Pik3c3* mRNA is universally expressed in all types of tissues in mammals [33]. Consistent with this, our in situ hybridization shows ubiquitous expression of *Pik3c3* in all cells of the developing embryos (Figure 1A1). Our lab previously generated a conditional allele of *Pik3c3* in which the ATP binding domain of the kinase is flanked by two *LoxP* sites (*Pik3c3^flox/flox^* mice) and thus can be deleted in the presence of Cre recombinase to result in a functional null allele [31]. We generated heterozygous mice (*Pik3c3^+/-^*) by crossing the conditional allele (*Pik3c3^flox/flox^*) with *Meox-cre* transgenic mice in which Cre is expressed in the germ line [34]. The resulting (germline deletion) null allele was subsequently introduced into C57BL/6J mice background through back crossing. Heterozygous *Pik3c3^+/-^* mice are fertile, phenotypically comparable to the wild-type controls and can live at least up to 18 month. We observed neither obvious behavioral defects nor spontaneous tumor genesis in heterozygous mice during this period.

### Early embryonic lethality in *Pik3c3* mutation

*Pik3c3* homozygous mutants (*Pik3c3* ^-/-^) were obtained by intercrosses between the heterozygous mice. Of the 64 P0 (postnatal day 0) offsprings from the intercrosses examined, 42 were heterozygous and 22 were wild-type, but no viable *Pik3c3* ^-/-^ pups were ever identified, indicating that homozygosity for the *Pik3c3* mutation leads to embryonic lethality. To identify the timing of lethality, embryos at the stages of E7.5, E8.0, E8.5 and E10.5 from heterozygous intercrosses were collected and genotyped by PCR (Figure 1A2). Homozygous embryos with a correct Mendelian ratio were recovered at E7.5 (Figure 1A3). Between E7.5 and E8.5, absorbed debris or empty deciduae were frequently observed and the number of viable homozygous mutants decreased (Figure 1A3). No homozygous mutant embryos were obtained at or after E8.5 (Figure 1A3). The results indicate that PIK3C3 is essential for embryogenesis at the initiation of gastrulation.

### Histological analysis of *Pik3c3* ^-/-^ embryos

**Figure 2:**
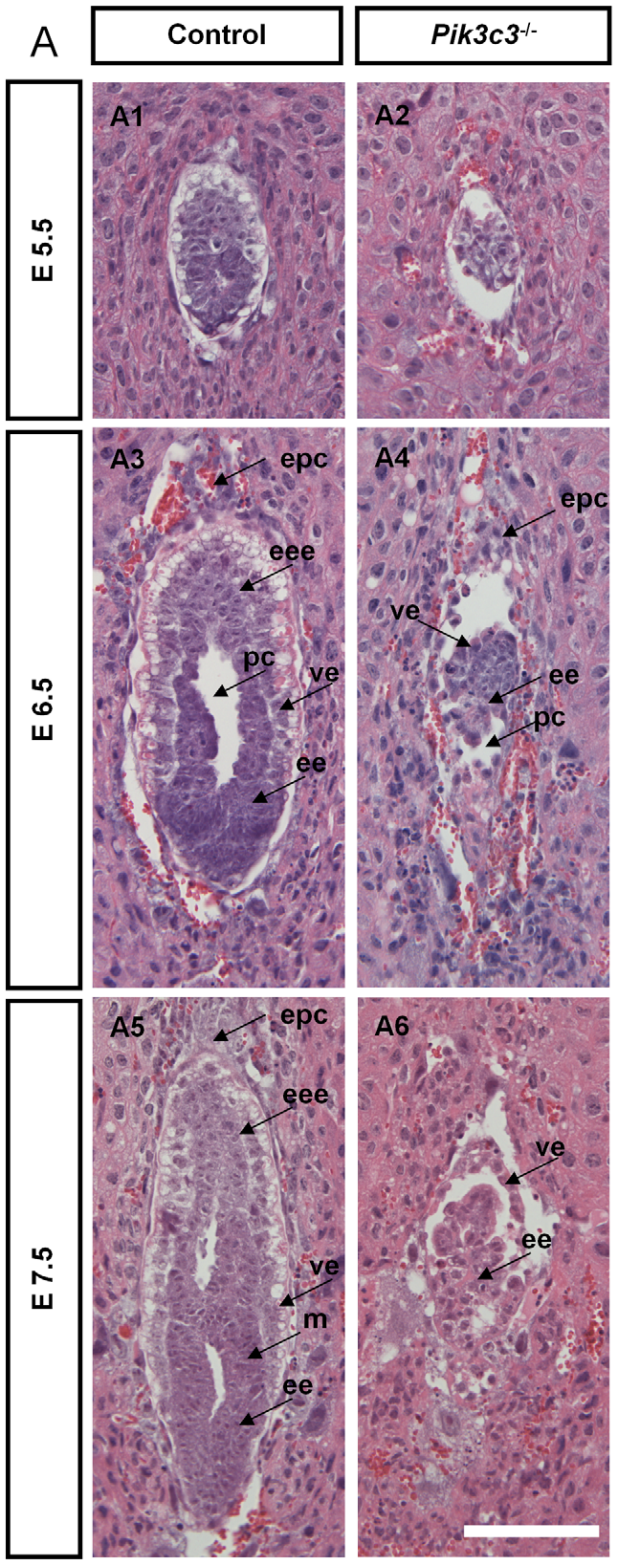
Histological analysis of *Pik3c3* mutant embryos. **A.** Representative images of Hematoxylin & Eosin (H&E) staining on control and *Pik3c3* deleted embryo sections at E5.5, E6.5 and E7.5. Arrows in E6.5 and E7.5 embryo point to ectoplacental cone (epc), extraembryonic ectoderm (eee), embryonic ectoderm (ee), mesoderm (m), proamniotic cavity (pc), visceral endoderm (ve). The mutant embryos are disorganized, smaller in size and have much less number of cells compared with controls. Scale bars: 100 µm.

To examine the phenotypes prior to embryo death, whole embryos were dissected out at E7.5. Heterozygous null embryos and wild-type controls are comparable and equally well developed at this stage (Figure 1B1). However, the homozygous mutant embryos remain the shape of a compact egg and are less than half the size of the wild-type and heterozygous embryos (Figure 1B2). Both embryonic and extra-embryonic regions in mutant embryos are smaller than controls, suggesting abnormal embryogenesis (Figure 1B1 and 1B2). To examine the mutant phenotypes in further detail, we performed H&E staining of serial sagittal sections of E5.5, E6.5 and E7.5 embryos (Figure 2). At E5.5 egg cylinder–stage, the mutant embryos can be readily distinguished by their smaller size (Figure 2A1 and 2A2). At E6.5, control embryos exhibit well-organized visceral endoderm (ve), embryonic ectoderm (ee), a proamniotic cavity (pc) and a forming mesoderm (m) that is more obvious at E7.5. By contrast, mutant E6.5 embryos do not appear to progress much beyond E5.5 (Figure 2A3 2A4 and 2A5; arrows). They contain less identifiable layers of visceral endoderm (ve) and embryonic ectoderm (ee), and are much smaller in size and have much less number of cells compared with controls. Mutant embryos exhibit no detectable primitive streak and some contain a small proamniotic cavity (pc) (Figure 2A3 and 2A4; arrows). At E7.5, mutant embryos grow slightly in size. A layer of cells that appear to be visceral endoderm (ve) can be seen surrounding the embryo, but the rest of the embryos are disorganized and it is difficult to discern any structures (Figure 2A5 and 2A6; arrows). These data indicate that the development of *Pik3c3^-/-^* embryos arrests at the beginning of gastrulation.

### *Pik3c3* mutant embryos lack mesoderm

**Figure 3:**
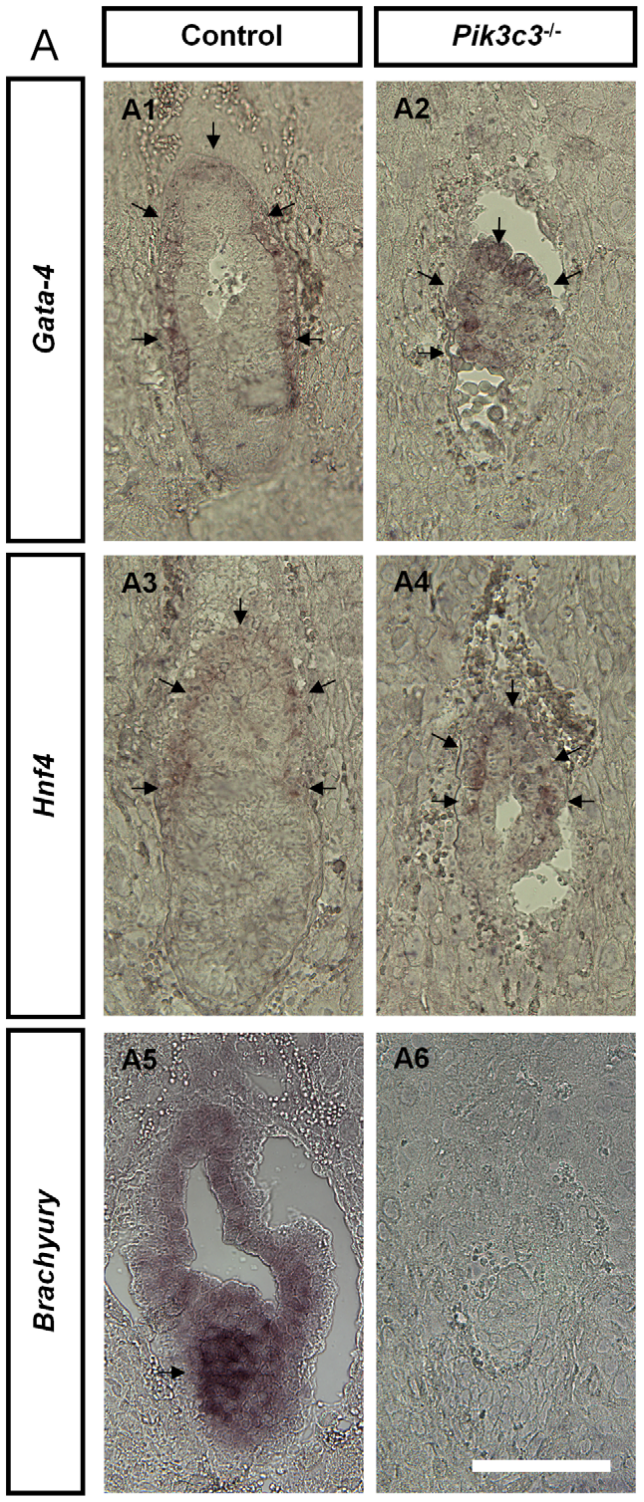
*Pik3c3* mutant embryos lack mesoderm. **A.** Representative images of in situ hybridization of *Gata4*, *Hnf1* and *Brachyury* in control and *Pik3c3* mutant embryo sections at E6.5. Both *Gata4* and *Hnf1* label visceral endoderms in control and mutant. No *Brachyury* labeling was observed in mutant embryos. Scale bars: 100 µm.

We next examined the expression of molecular markers for different embryonic layers at E6.5. Visceral endoderm in wild-type embryos expresses transcription factors *Hnf4* (hepatocyte nuclear factor-4) [35], [36] and *Gata4* [37], [38] (Figure 3A1 and 3A3). Both markers also label a thin layer of cells covering the mutant embryos (Figure 3A2 and 3A4), suggesting that mutant embryos indeed possess visceral endoderm. On the other hand, morphological and histological studies mentioned above implicate a missing mesoderm in mutant embryos. Consistent with this, the transcript factor *Brachyury* that labels primitive streak at E6.5 in wild-type animals [39] (Figure 3A5) is completely missing in mutant embryos (Figure 3A6). The results further confirm that mutant embryos fail to form mesoderm.

### *Pik3c3* mutation causes cell proliferation defects *in vivo*

**Figure 4:**
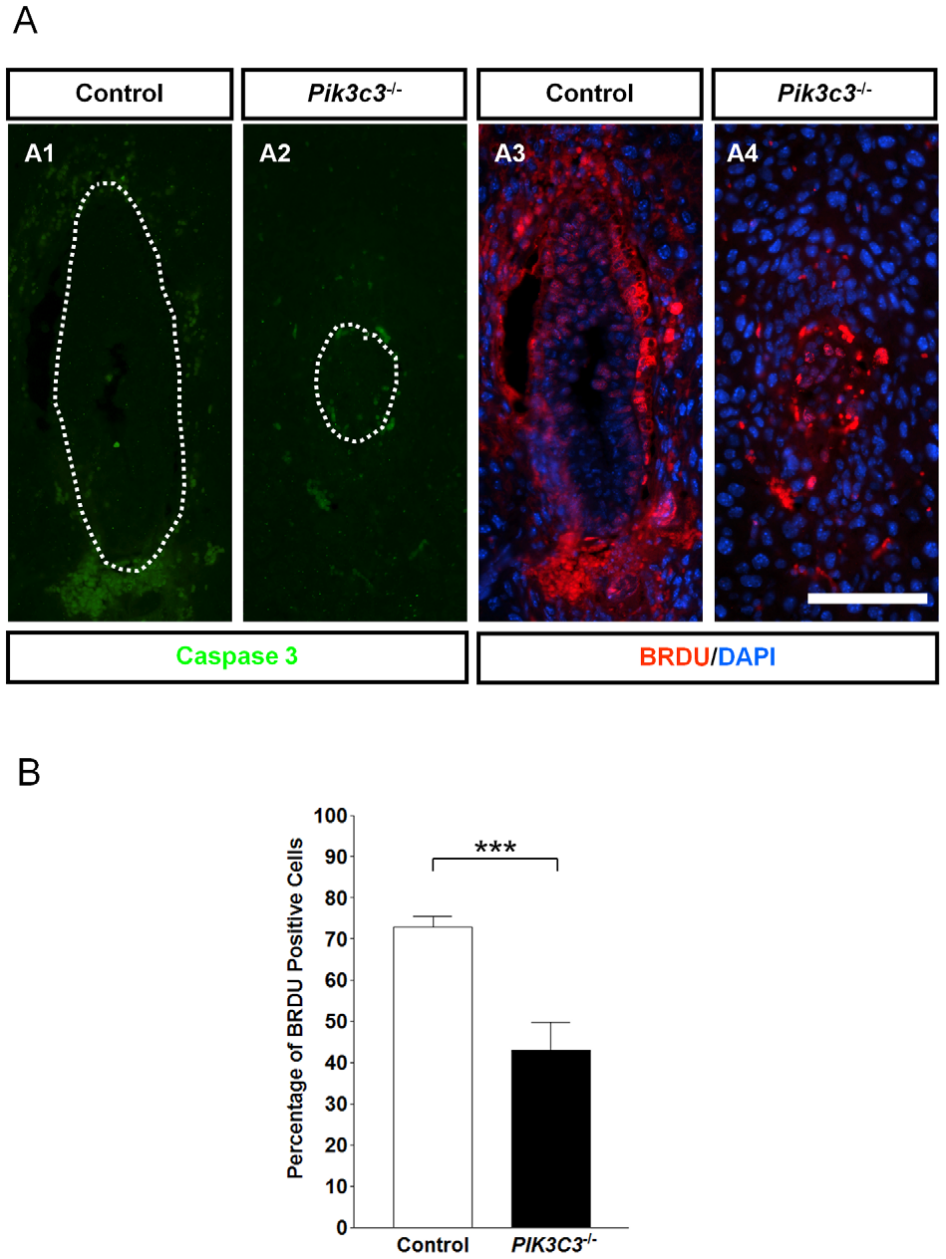
No apoptotic cell death but reduced proliferation in *Pik3c3* mutant embryo. **A.** Activated caspase 3 immunostaining (green) and BrdU labeling (red) in control and mutant embryos at E6.5. Scale bar: 100 µm. **B.** Bar graph shows percentage of BrdU positive cells over total number of cells in control and mutant embryos at E6.5. Wild type embryos show an average ratio around 0.7 while mutant embryos show an average ratio around 0.4 (p<0.001).

The growth defects and reduced cell numbers in *Pik3c3* mutant embryos could result from either an excess of programmed cell death or a reduced cell proliferation rate. To distinguish between these two possibilities, we first examined apoptosis. E6.5 control and mutant embryos were serially sectioned and stained with antibody against active Caspase 3 (Figure 4A1 and 4A2). Cells in both control and mutant embryos lack positive Caspase 3 signal (Figure 4A1 and 4A2). The lack of apoptosis is further confirmed by electron microscopic analysis of E6.5 embryos, in which we did not detect any morphological alterations associated with apoptotic cell death, such as condensed and fragmented nuclear chromatin or interrupted nuclear envelope, for either of the genotypes (see below the ultra-structural analyses part). These results suggest that there is no excessive apoptosis occurring in *Pik3c3* mutant embryos.

Next, we examined the effect of the *Pik3c3* mutation on cellular proliferation *in vivo* by BrdU incorporation. BrdU was injected into pregnant females at E6.5 and allowed to label proliferating cells for one hour (Figure 4A3 and 4A4). The ratio of the number of BrdU-positive nuclei to the total number of cells (as indicated by DAPI staining) was calculated to serve as the proliferation index (Figure 4B). Many cells in wild-type embryos were BrdU-positive, producing an average proliferation index of 72.8% (with a standard error of the mean, or SEM, of 2.6%) (Figure 4A3 and 4B). However, the mutant embryos analyzed showed an average index of only 43.1% (with a SEM of 6.7%) (Figure 4A4 and 4B), suggesting a significantly impaired cell proliferation capacity in mutant embryos *in vivo*. Therefore, reduced proliferation, but not excessive cell death, is likely to account for the small size and retarded growth of *Pik3c3* mutant embryos.

### *Pik3c3* mutation causes cell proliferation defects *in vitro*

**Figure 5:**
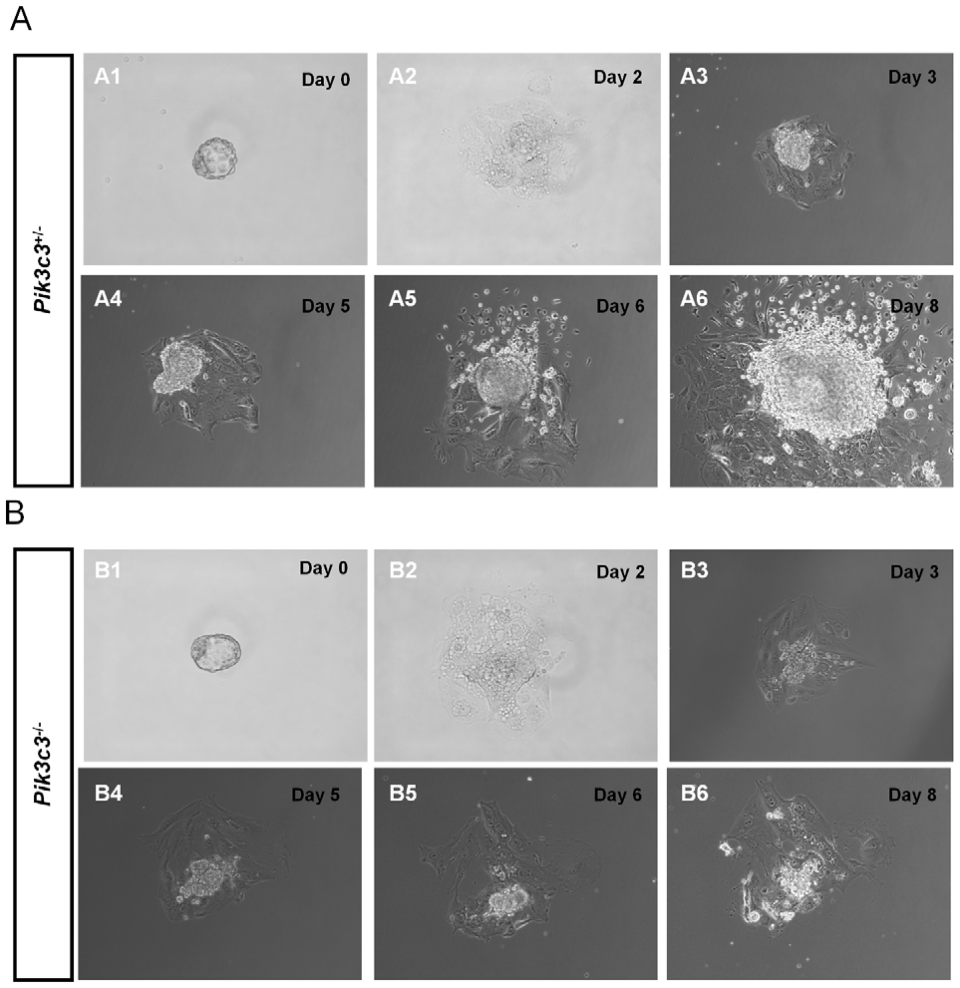
*Pik3c3* deleted blastocysts show defects in cell proliferation in culture. **A.** Representative images of cultured blastocysts from a heterozygous control embryo. Embryos were collected from heterozygous intercrosses at E3.5 and genotyped after cultured *in vitro* for 8 days. Inner cell mass (ICM) and trophoblast giant cells (TGC) are indicated at Day 8. **B.** Representative images of cultured blastocysts from a homozygous mutant embryo. Mutant embryos have defects in proliferation.

To directly test the growth capacity of mutant *Pik3c3* embryos, we performed *in vitro* blastocysts growth assay. Blastocysts were collected from E3.5 embryos, cultured and imaged *in vitro* for 8 days followed by genotyping. Initially, all blastocysts have similar appearance, indicating that *Pik3c3* mutation does not affect pre-implantation development (Figure 5A1 and 5B1). During the first two days *in vitro*, both control and mutant blastocysts exhibited substantial trophoblast giant cell outgrowth and inner cell mass growth (Figure 5A2 and 5B2). Subsequently, however, the growth of both trophoblast giant cells and inner cell mass in all mutant blastocysts (n = 6) slowed down significantly compared with controls. In fact, there was very limited increase in cell numbers in the mutant cultures between day 3 and day 8 (Figure 5A3–A6, 5B3–B6). By contrast, the inner cell mass from all control blastocysts (9 heterozygous, 5 wildtype) proliferated substantially and formed big colonies after 8 days of culture. These results suggest that even with nutrients provided in culture, *Pik3c3* mutant embryonic cells still proliferate poorly.

### Ultra-structural analyses of *Pik3c3* deficient visceral endoderm and epiblast cells

**Figure 6:**
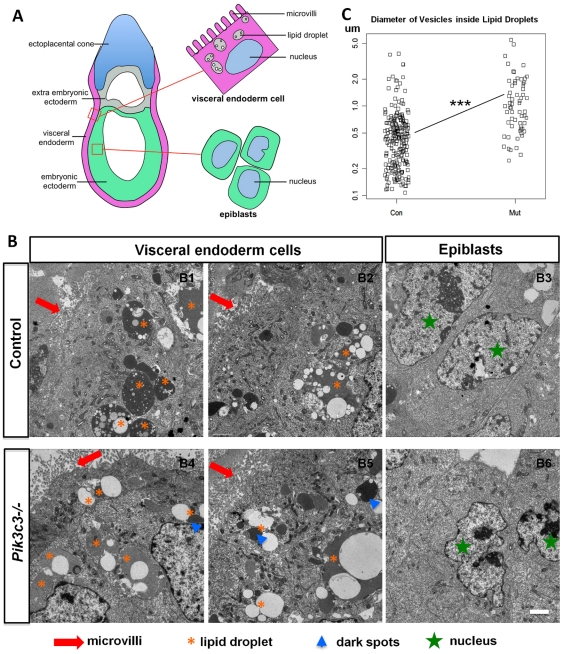
Ultrastructural analyses of *Pik3c3* mutant embryos. **A.** Schematic representation of locations of visceral endoderm cells and epiblasts of E6.5 embryos. Boxed regions show where EM images are taken. Microvilli, lipid droplets and cell nucleus are illustrated. **B.** Representative EM images of control and mutant embryonic cells at E6.5. B1-2 (control) and B4-5 (mutant) are high magnification EM images of visceral endoderm cells. Microvilli (red arrows) and lipid droplets (orange stars) are indicated. Notice the increases in size of clear vesicles inside the lipid droplets in mutant embryos. Most lipid droplets appear only light stained with only a few dark-stained spots in mutants (blue arrowheads). B3 (control) and B6 (mutant) are high magnification EM images of epiblast cells in embryonic ectoderms. Green stars point to nucleus. No differences in cell morphology or subcellullar organelles were observed in mutant epiblasts. Scale bars: 2 µm. **C.** Quantification on the diameter of vesicles inside lipid droplets, with scatter plot of both control and mutant embryos. There is a statistically significant increase in vesicle size inside the lipid droplets in mutant embryos (p<0.001).

Since PIK3C3 plays a critical role in the later steps of the endocytic pathway [4], and previous work showed that loss of PIK3C3 function results in an accumulation of enlarged vesicles inside cells [14], [15], [31], we wanted to examine whether loss of *Pik3c3* causes similar subcellular defects in embryos. We performed electron microscopy (EM) studies on E6.5 embryos. Figure 6A is a schematic representation of the regions in the embryo where EM images were taken. We first examined visceral endoderm cells, which are responsible for uptaking and transporting nutrient to support the rapid proliferation and growth of the epiblasts during the initiation of gastrulation [40], [41], [42]. In both control and mutant embryos, the visceral endoderm cells surrounding the developing embryos exhibit a well-defined brush border composed of dense apical microvilli (Figure 6A, 6B1-2, 6B4-5; red arrows). No apparent differences were found for these microvilli structures. Inside the visceral endoderm cells, there is a subtle but observable change in the morphological organizations of the internalized lipid droplets. In the control visceral endoderm cells, numerous small clear vesicles can be observed inside the dark lipid droplets (Figure 6B1-2, orange stars mark lipid droplets), with an average diameter of 0.55 um (and a SEM of 0.04 um) (Figure 6C). In mutant embryos, the size of the vesicles inside lipid droplets is significantly increased, with an average diameter of 1.28 um (and a SEM of 0.13 um) (Figure 6B4-5 and 6C), and most of lipid droplets appear only lightly stained with only a few dark-stained spots (Figure 6B4-5; blue arrowheads). These morphological differences suggest the possibility that either the processing or the transportation of internalized lipids and fluids might be subtly altered in mutant embryos. We next examined the epiblast cells which are cells give rise to the embryo (Figure 6A). Epiblasts are in the process of rapid growth and dividing, and they have somewhat irregularly shaped nuclei (Figure 6B3, 6B6; green stars). We found no obvious ultrastructural differences between control and mutant epiblasts (Figure 6B3 and 6B6). This result suggests that there is probably only limited endocytosis going on in the proliferating epiblasts, and thus no overt abnormal endosome structures are observed in these cells.

### Reduced mTOR signaling in *Pik3c3* mutation embryos

**Figure 7:**
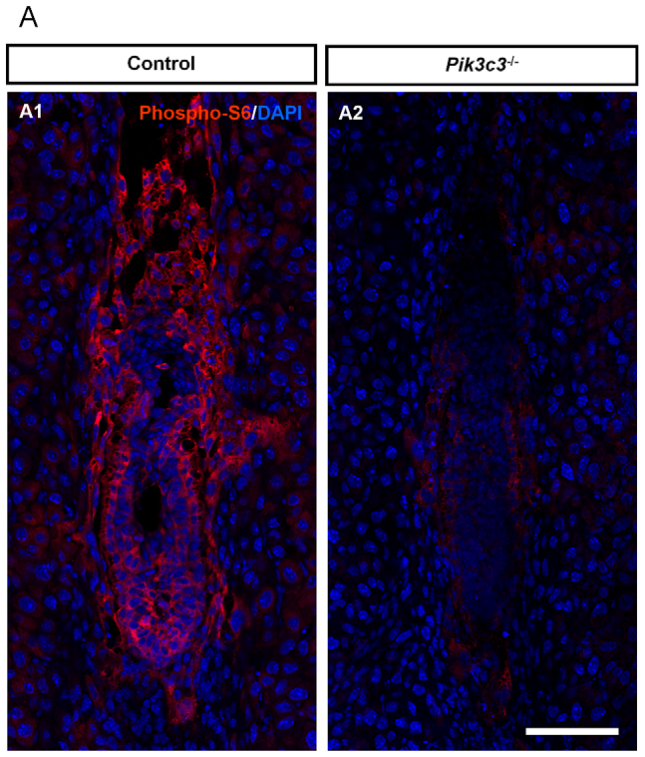
Significantly reduced Phospho-S6 level in *Pik3c3* mutant embryos. **A**. Immunostaining of phosphor-S6 (red) in control and mutant embryos at E6.5, counter stained with DAPI (blue). Notice the drastically decreased phospho-S6 level in mutant embryos. Scale bar: 100 µm.

If an endocytic process does not contribute significantly to the proliferating defects of *Pik3c3* embryos, what other PIK3C3 regulated processes might cause the failed growth? PIK3C3 has been shown to regulate nutrient sensing and amino acid induced mTOR activation in cultured cells [23], [24], [25], [26]. mTOR mutant embryos show similar early embryonic lethality and defects in cell proliferation as the *Pik3c3* mutant mice [43], [44], [45]. On the other hand, growth factor signaling through activation of Akt pathway can also lead to mTOR activation. Thus it is not immediately clear whether mTOR signaling is affected in *Pik3c3* mutant embryos. We performed immunostaining of phosphorylated ribosomal protein S6 (phospho-S6), the downstream target of mTOR complex 1 and S6 kinase 1 (S6K1) and also a commonly used indicator for mTOR activation, in E6.5 control and mutant embryos [45]. Strong phospho-S6 staining can be clearly visualized in control embryos (Figure 7A1), indicating a highly active mTOR signaling pathway during early embryogenesis. In contrast, phospho-S6 level is drastically reduced in *Pik3c3* mutant embryos (Figure 7A2). This result is consistent with previous findings that during earliest stages of development, the embryo is very much dependent on branched chain amino acids and glucose, rather than growth factors, to activate mTOR signaling for growth [46], [47], [48]. Thus, the loss of mTOR signaling in *Pik3c3* mutant embryos could be a major contributor to the observed cell proliferation defects and early embryonic lethality.

## Discussion

In the present study, we generated *Pik3c3* knockout mice and characterized the *in vivo* function of PIK3C3 during embryogenesis. The most prominent defect caused by *Pik3c3* deletion is a severely reduced embryonic cell proliferation. The death of *Pik3c3* mutant embryos prior to gastrulation is likely due to the lack of proliferative burst required for the development of different germ layers. It has been shown that mouse gastrulation occurs at around E6.5 when mesoderm is generated from epiblast [42]. Importantly, there is normally a ∼100-fold increase in cell number between E5.5 and E7.5 [42]. The dramatic increase in cell proliferation is necessary for proper gastrulation since cells need to accumulate to certain numbers to initiate this process [49]. In fact, mutations in genes that affect proliferation rate during this period often result in missing of mesoderm, abnormal embryonic development and early embryonic lethality [39]. For instance, embryos carrying null mutation in genes encoding proteins of the bone morphogenetic protein (BMP) signaling pathway, including *Smad4* [50], [51], *Bmp4* [52] and *Bmpr* [53], all fail to develop mesoderm and die at the beginning of gastrulation partly due to defects in cell proliferation. Germline deletion of tumor suppressor genes regulating cellular growth such as *Brca1* [39] and *Tsg101* [54] also leads to cell proliferation defect, lack of mesoderm and early embryonic lethality before E7.5. Here we show that *Pik3c3* mutation leads to significantly retarded cell proliferation both *in vivo* and *in vitro*. Interestingly, the blastocysts isolated from E3.5 *Pik3c3* mutant embryos appear normal compared with controls and display similar growth and proliferation capability in the first two days in culture. It is possible that persistence of maternal PIK3C3 protein or mRNA may permit cell proliferation and the initial development of blastocysts. Subsequently, as PIK3C3 proteins are gradually depleted, the growth of embryos stops.

Previous studies have established critical roles of PIK3C3 in autophagic and endocytic pathways [4]. Since knockout of genes essential for autophagy, *Atg5* or *Atg7*, results in normal embryonic development and postnatal lethality [55], [56], autophagy alone is unlikely to contribute to the phenotypes observed in *Pik3c3* mutant embryos. On the other hand, we found somewhat altered lipid uptake/transportation in visceral endoderm cells in *Pik3c3^-/-^* embryos, which could potentially contribute to the significantly reduced proliferation *in vivo*. Visceral endoderm cells function in nutrient uptake, to support rapid cell proliferations during early development [40]. Amino acids are taken into these cells by transporter proteins, whereas lipids are internalized through endocytosis. In vitro, the endoderm surrounding the inner ectoderm core is also required for the proliferation of inner cell mass [43], [57]. Deletion of several key genes that control visceral endoderm differentiation, including *Gata4* [58], [59], *Gata6* [60] and *Hnf4* [61], all lead to early developmental arrest. Moreover, knockout of the gene encoding the adaptor protein Dab2 which regulates megalin endocytosis in the visceral endoderm cells results in early embryonic lethality and cell proliferation arrest both *in vivo* and *in vitro* [62], [63], [64]. EM analysis of *Pik3c3* null embryos revealed a mild accumulation of enlarged vesicles inside lipid droplets in mutant visceral endoderm cells. This phenotype is consistent with the role of PIK3C3 in regulating late steps of endocytic pathway [14], [15], [31]. This ultrastructural change is specific to visceral endoderm cells but not to cells inside the embryo proper, in agreement with the fact that endocytosis is particularly active in visceral endoderm cells. However, further experiments are needed to test whether the subtly altered vesicle sizes in lipid droplets indeed affect lipid transportation into the embryo proper.

Finally, our study also provides *in vivo* evidence that mTOR signaling is significantly reduced in *Pik3c3* mutant embryos. Two distinct mTOR signaling complexes exist in mammals [65]. The complex 1 contains mTOR, raptor and other proteins, and is activated by nutrients. The complex 2 contains mTOR, rictor and other components, and is activated by the growth factor mediated Akt/PKB pathway [65]. Interestingly, knockout of genes encoding mTOR or the complex 1 component raptor causes a cell proliferation defect and early embryonic lethality at E6.5, a phenotype closely resembles that in *Pik3c3* knockout mice [43], [44], [45]. By contrast, deletion of the gene encoding the complex 2 component rictor only results in death at a much later developmental stage [45]. These results suggest that mTOR is primarily activated by the nutrient pathway in early embryos, consistent with the fact that branched amino acid chains support early embryonic growth [46], [47], [48]. Importantly, recent cell culture studies revealed a critical role of PIK3C3 in nutrient sensing and activation of mTOR complex 1 pathway. It has been shown that amino acids can induce a rise in intracellular Ca^2+^ that activates a direct binding of calmodulin to PIK3C3 [23]. This binding activates the lipid kinase activity of PIK3C3, which is required for the activation of mTOR complex 1 and the subsequent activation of S6K1 [24], [26]. Our study now provides the *in vivo* evidence that amino acid activated mTOR signaling is indeed drastically reduced in *Pik3c3* mutant embryos. This finding also suggests that the cell proliferation defects and failed embryogenesis in *Pik3c3* mutant is at least partially due to the loss of mTOR signaling.

## Materials and Methods

### Generation of *Pik3c3-KO* Mice

Mice heterozygous for the conditional allele (*Pik3c3^flox/+^*) [31], [32] were bred with mice expressing a *Meox-cre* transgene with cre expressed in the germ line to generate *Pik3c3* ^+/−^ animals. Note that in some cases, Meox-Cre gave rise to incomplete (mosaic) deletion of the *Pik3c3^flox^* allele, so the offsprings are further back crossed with C57/B6 mice to obtain *Pik3c3^+/-^* heterozygous mice on the C57/B6 background. Homozygous mutant mice (*Pik3c3* ^-/-^) were obtained by heterozygous intercrosses. PCR based strategies were used for genotyping and the following PCR primers were used to detect *Pik3c3^-^* (deleted) allele: *L1, 5′- AACTGGATCTGGGCCTATG-3′; L2 5′-GAAGCCTGGAACGAGAAGAG-3′; L3, 5′-CACTCACCTGCTGTGAAATG-3′.* The deleted allele produces a 670 bp fragment while the wild type allele produces a 450 bp fragment. All experiments were conducted according to protocol #A267-09-09, approved by the Duke University Institutional Animal Care and Use Committee.

### Blastocyst Cultures and Genotyping

Embryonic stage (days postcoitum) was estimated by timed pregnancy. E3.5 embryos from *Pik3c3* **^+/-^** heterozygous intercrosses were collected by flushing through the uterus of the plugged females. Blastocysts were individually collected with mouth pipettes and cultured in 12-well plates in 5% CO_2_ incubator at 37°C. Culture medium is the standard embryonic stem cell (ES cell) culture medium without leukemia inhibitory factor (10%FBS, 2 mM L-glutamine, 0.1 mM non essential amino acid, 1 mM sodium pyruvate, 1∶100 penicillin/streptomycin and 50 µM β-mercaptoethanol in 1x GMEM). Photos were taken every 24 hr. After 8 days *in vitro*, cultured blastocysts were lysised overnight at 55°C in 100 ul of lysis buffer (50 mM KCl, 10 mM Tris (pH 8.3), 2 mM MgCl_2_, 0.1 mg/ml gelatin, 0.45% NP-40, and 0.45% Tween-20) containing 200 µg/ml proteinase K. Samples were boiled for 10 min afterwards. 1-5 µl of samples were subjected to PCR amplification with 40 cycles to determine the genotypes. A total of 20 blastocysts obtained from two pregnant females were cultured and genotyped, among which 5 were confirmed to be wild-type controls, 9 were heterozygous and 6 were homozygous mutants.

### Histological Analyses

#### Fixation and embedding

Plugged females at the desired stage were sacrificed. The uteri were removed and the decidual swellings were fixed in 4% paraformaldehyde at 4°C overnight. They were either embedded in OCT, or dehydrated and embedded in paraffin. 16–18 µm sections were cut from OCT embedded samples and 5–7 µm sections were cut from paraffin embedded samples.

#### In situ hybridization

In situ probe templates were generated by RT-PCR from embryonic tissues. Specifically, Pik3c3 probe was amplified with *5′-ACCACGATCTCAAACCCAACGCTAC-3′* and *5′-AGGGTAGCTGTTTCCGGGATGATGC-3′*; the *Brachyury* probe which labels mesoderm was amplified with *5′-TGACCAAGAACGGCAGGAG-3′* and *5′-GAACCAGAAGACGAGGACG-3′*; the *Hnf-4* probe was amplified with *5′-ACACGTCCCCATCTGAAGGTG-3′* and *5′-CTTCCTTCTTCATGCCAGCCC-3′*; the *Gata-4* probe was amplified with *5′-GGCTGTCATCTCACTATGG-3′* and *5′-TGGGGTGAAGGAGATTATGTG-3′*. DIG labeled antisense probes were synthesized by *in vitro* transcription using T7 RNA-polymerase (Roche) with Digoxigenin (DIG)-UTP (Roche). In situ hybridization was performed according to standard methods. 3 embryos from each group (control or mutant) were used for each molecular marker labeling, and 2 wild-type embryos from each developmental stage (E6.5 or E7.5) were used for in situ hybridization of *Pik3c3*.

#### Immunostaining

The following antibodies were used: rabbit anti-caspase 3 active (R&D Systems, 1∶500), rabbit anti-phospho-S6 ribosomal protein (ser235/236, Cell Signaling, 1∶100), DAPI (Sigma, 1∶5000), Alexa Fluor 488- and 568-conjugated goat anti-rabbit IgG (Invitrogen, 1∶400), Alexa Fluor 568-conjugated goat anti-mouse IgG (Invitrogen, 1∶400). Standard immunofluorescence procedure was used as described before [66]. Briefly, sections were incubated with 1° antibody at 4°C overnight, washed with 1X PBS three times for 10 min each, incubated with 2° antibody at room temperature for 2 hr, and finally washed with 1X PBS three times for 5 min each. 2 to 10 embryos from each group (control or mutant) were used for immunostaining.

#### H&E staining

Hematoxylin and eosin staining was performed according to standard protocol. 2–3 embryos from each group (control or mutant) at each different embryonic stage (E5.5, E6.5 or E7.5) were used. Different embryonic structures were identified by location.

#### BrdU labeling

Bromo-deoxyuridine (BrdU) labeling reagent (GE Health, 2 ml/100 g) was IP injected into plugged heterozygous female with E6.5 embryos. After one hour, all embryos from the litter were collected, fixed, embedded in paraffin, and sectioned with a microtome. Sections were incubated in 2N HCl at 37°C for 2 hr, treated with 0.05% Trypsin in PBS at room temperature for 2 min, and were finally stained with monoclonal anti-BrdU antibody (Sigma, 1∶500). Embryo boundaries were identified by both BrdU staining and bright field image. The number of BrdU positive cells in embryos on each section was counted and normalized with respect to the total number of cells to yield a proliferation index. N = 7–11 sections from 3 different embryos of the same genotype (control or mutant) were used for quantification and the *p-value* was calculated using standard Student's t-test.

### Electron microscopy

Litters of E6.5 embryos from *Pik3c3^+/-^* heterozygous intercrosses were collected and fixed for electron microscopy studies. Tissues were perfused and fixed with 2% paraformadehyde+2.5% glutaraldehyde (Polysciences, Inc) in 0.1M sodium cacodylate buffer pH 7.4 (Electron Microscopy Sciences) for 3 days, washed with PBS, fixed with 1% osmiumtetroxide (Electron Microscopy Sciences)+1.5% potassiumferrocyanide (Electron Microscopy Sciences) in H_2_O for 2 hr, washed with maleate buffer (Electron Microscopy Sciences), dehydrated in 70%, 90% and 100% ethanol 15 min each, dehydrated again in propyleneoxide (Electron Microscopy Sciences) for 1 hr, infiltrated in 50% Epon (TAAB+DDSA+NMA+DMP-30, Canemco & Marivac)+50% propyleneoxide for 3 hr, all at room temperature. Tissues were eventually embedded in Epon for 2 days at 60°C. Semi-thin and Ultra-thin sections were cut using a Reichert Ultracut S microtome. Semi-thin sections of all embryos were first cut and stained with toluidine blue solution (0.2% toluidine blue O and 1% sodium chloride, pH 2.3) to identify which embryos are mutants and which embryos are controls, and to find the region where the embryo is located. All mutant embryos show similar disorganized and smaller appearance. Subsequently ultra-thin sections were cut and observed with a JEOL 1200 EX transmission electron microscope.

The diameters of all the vesicles inside each lipid droplets in 6–7 randomly selected ecdoderm cells from 2 controls and 2 mutant embryos were measured with Metamorph and the *p-value* was calculated using standard Student's t-test.
